# Screening of Excitons by Organic Cations in Quasi-Two-Dimensional Organic–Inorganic Lead-Halide Perovskites

**DOI:** 10.1021/acs.nanolett.2c01306

**Published:** 2022-06-09

**Authors:** Marina R. Filip, Diana Y. Qiu, Mauro Del Ben, Jeffrey B. Neaton

**Affiliations:** † Department of Physics, University of Oxford, Clarendon Laboratory, Oxford OX1 3PU, United Kingdom; ‡ School of Engineering and Applied Science, Yale University, New Haven, Connecticut 06511, United States; ¶ Computational Science Division, Lawrence Berkeley National Laboratory, Berkeley, California 94720, United States; § Department of Physics, University of California, Berkeley, California 94720, United States; ∥ Materials Science Division, Lawrence Berkeley National Laboratory, Berkeley, California 94720, United States; ⊥ Kavli Energy Nano Sciences Institute at Berkeley, Berkeley, California 94720, United States

**Keywords:** layered perovskites, excitons, screening, halide perovskites, quantum confinement, optical properties

## Abstract

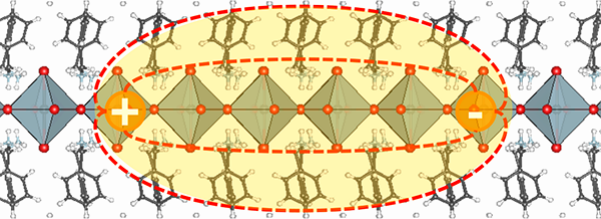

Interlayer organic cations in quasi-two-dimensional halide perovskites are a versatile tuning vehicle for the optoelectronic properties of these complex systems, but chemical intuition for this design route is yet to be established. Here, we use density functional theory, the *GW* approximation, and the Bethe-Salpeter equation approach to understand the contribution of the organic cation to the quasiparticle band gap and exciton binding energy of layered perovskites. We show that organic cations in quasi-two-dimensional perovskites contribute significantly to the dielectric screening in these systems, countering quantum confinement effects on the quasiparticle band gap and the exciton binding energy. Using a simple electrostatics model inspired by parallel-plate capacitors, we decouple the organic cation and inorganic layer contributions to the effective dielectric constants and show that dielectric properties of layered perovskites are broadly tunable via the interlayer cation, providing a direct means of tuning photophysical properties for a variety of applications.

Layered hybrid organic–inorganic metal-halide perovskites are a highly promising family of quasi-low-dimensional heterogeneous semiconductors for optoelectronic applications.^1−8^ These materials are generally highly stable in ambient conditions and can be flexibly synthesized in bulk form,^4,6^ exfoliated to a monolayer,^9^ assembled in interfaces with transition metal dichalcogenides^10,11^ or other layered perovskites,^12,13^ or self-assembled in perovskite-nonperovskite single-crystal heterostructures.^14^ The structural and chemical versatility of layered perovskites is in large part due to the wide range of organic cations that may be incorporated between the perovskite layers.^8^ However, this highly attractive feature significantly enhances their structural and chemical complexity and poses great challenges for the fundamental study of their optoelectronic properties.

Layered lead-halide perovskites can be thought of as derivative structures of the three-dimensional (3D) halide perovskites (e.g., CH_3_NH_3_PbI_3_^15,16^). Depending on the size, shape, and charge of the organic cation, layered perovskites can arrange in either flat layers of corner-sharing inorganic octahedra, more complex corrugated layers, or layers which include both corner and face-sharing octahedra.^4,6,8,14^ Ruddlesden–Popper (RP) perovskites have a standard chemical formula of A′_*n*–1_A_2_B_*n*_X_3*n*+1_, where A and A′ are monovalent cations, B is a divalent metal cation, X is a halogen anion, and *n* is the number of BX_6_ octahedra stacked up in a single layer.^4^ In the *n* = 1 limit, RP perovskites have a chemical formula of A_2_BX_4_ and exhibit an effectively 2D behavior, with electronic coupling between layers suppressed by organic cations.^17^ Quantum confinement effects in this case increase the quasiparticle band gap by more than 1 eV and the exciton binding energy by up to a factor of 10, as compared to 3D perovskite counterparts.^3^ By increasing *n* from the quasi-2D limit (*n* = 1) toward bulk (*n* = ∞), quantum confinement effects are gradually reduced, and the optical absorption onset and exciton binding energies are red-shifted.^18,19^

The optoelectronic properties of A_2_BX_4_-type perovskites are highly tunable via partial or total chemical substitution of the A, B, and X components, in close similarity to their 3D counterparts.^20−29^ The optical spectrum blue shifts with decreasing X anion^30^ and red shifts from Pb to Sn.^31^ As in 3D perovskites, the A-site cations do not contribute directly to optical transitions close to the onset.^29^ Furthermore, as in 3D perovskites,^29,32−36^ A-site cations in layered perovskites induce structural changes on the inorganic octahedral network, which, in turn, modulate the quasiparticle band gap and charge carrier effective masses.^30,37,38^ In contrast, while the A-site cation does not significantly impact the high frequency dielectric constant of 3D halide perovskites,^39,40^ and implicitly the binding energy of photoexcited charge carriers,^34,35^ experimental reports suggest that altering the organic cations in layered perovskites may reduce the exciton binding energy by up to 300 meV.^41−44^

Advances in density functional theory (DFT)^45^ and *ab initio* many-body perturbation theory (MBPT)^46,47^ within the *GW* approximation^48^ for single-particle excitations and the Bethe-Salpeter equation (BSE)^49,50^ approach for neutral two-particle excitations have enabled this framework to be successfully employed to study materials with increasing degrees of complexity, such as bulk and monolayers of transition metal dichalcogenides,^51−53^ organic semiconductors,^54^ and heterogeneous metal-halide perovskites.^39,55−57^ Prior *ab initio* calculations of the electronic structure of layered perovskites have so far been largely focused on understanding dielectric and quantum confinement effects driven by the inorganic layer thickness,^58,59^ shape,^17,60^ and the organic layer^18^ and limited to standard DFT approaches. Due to the structural complexity of layered halide perovskites, *GW*+BSE methods have only been employed in two prior studies focusing on understanding the optical spectra and exciton binding energy in RP perovskites with *n* = 1, 2^61^ and a model lead-halide perovskite monolayer with A = Cs.^62^ In addition, ref (63) used a tight-binding *GW*+BSE approach to calculate the exciton binding energies of RP perovskites with *n* up to 5, highlighting the nonhydrogenic nature of excitons in these compounds.

In this work, we perform *GW*+BSE calculations to understand the contribution from organic cations to screening photogenerated electron–hole pairs in three RP lead-bromide perovskite semiconductors with *n* = 1, BA_2_PbBr_4_ (BA = butylammonium), PMA_2_PbBr_4_ (PMA = phenylmethylammonium), and NMA_2_PbBr_4_ (NMA = naphthalenemethylammonium). We show that unlike in 3D perovskites, the organic cation makes a nontrivial contribution to the high frequency dielectric screening, which impacts both the quasiparticle band gap and the exciton binding energy. We quantify this contribution using a simple electrostatic model inspired by standard formulas for parallel and series capacitors and consistent with prior literature.^41,64^ We use this model to reveal how the organic cation can be designed to increase the effective dielectric constant of the layered perovskite, either by increasing the effective dielectric constant of the cation layer itself or by reducing the distance between the inorganic layers.

We start our study with PMA_2_PbBr_4_. In Figure 1b, we show the electronic band structure calculated using the Quantum Espresso code^65,66^ and the Perdew-Burke-Erzerhof parametrization of the generalized gradient approximation (DFT/PBE)^67^ including spin–orbit coupling (SOC),^100,200^ for the experimental crystal structure of PMA_2_PbBr_4_ as reported in ref (30) (see the SI for details). PMA_2_PbBr_4_ has a direct band gap at Γ, and PMA cations contribute to bands more than 1 eV away from the conduction and valence band edge states (see Figure 1b), in line with previous DFT calculations.^17,19^ Therefore, one could naively exclude any association of interlayer organic cations with the optical absorption onset of these systems.

**Figure 1:**
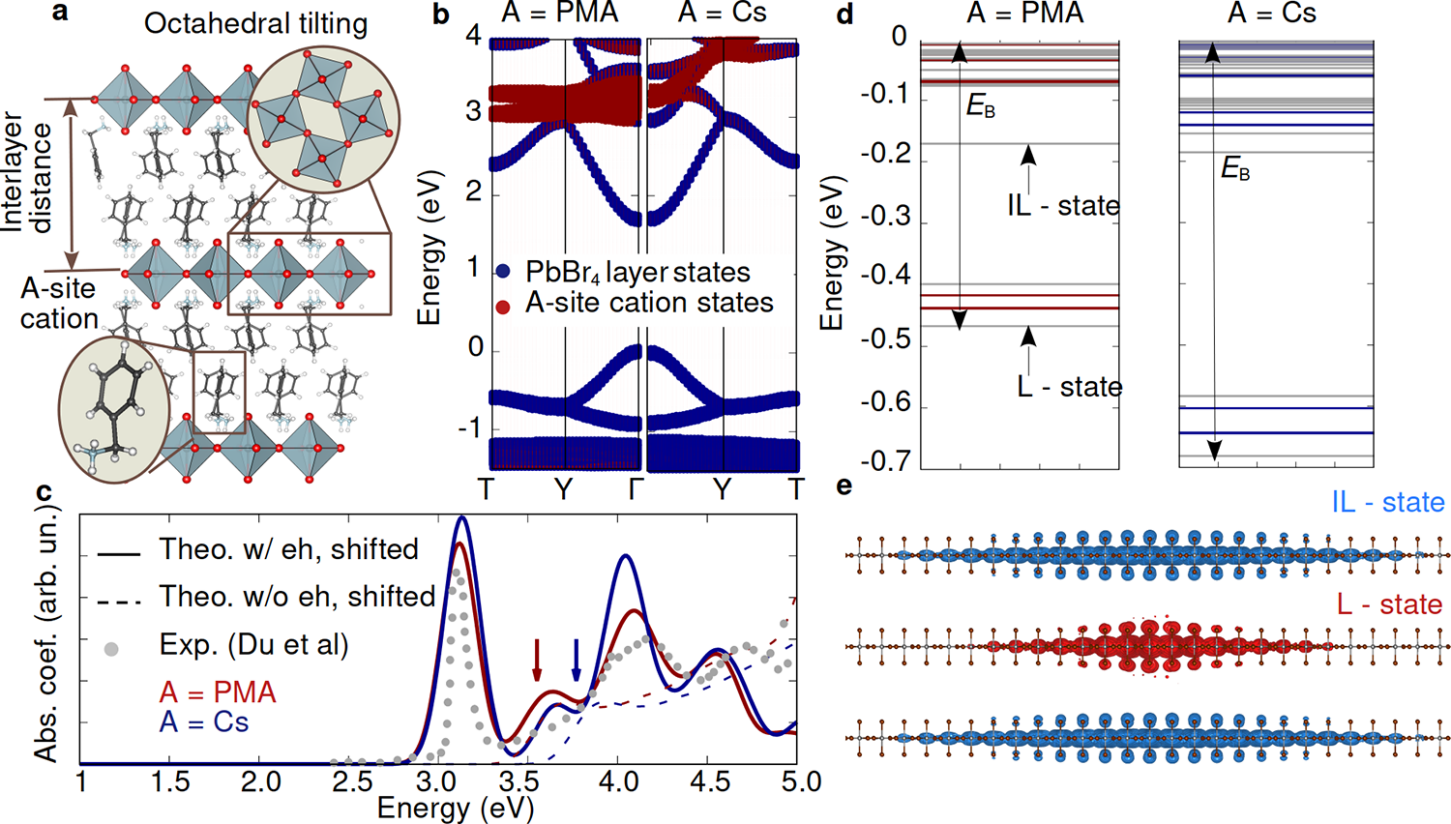
Summary of our DFT and *GW*+BSE calculations for PMA_2_PbBr_4_. **a.** Polyhedral model of the experimental PMA_2_PbBr_4_ reported in ref (30). **b.** Electronic band structure and projected density of states calculated within DFT/PBE including spin–orbit coupling for PMA_2_PbBr_4_ (left panel) and Cs_2_^PMA^PbBr_4_ (right panel). The zero of energy is set to the valence band edge. **c.** Optical absorption spectra calculated within the independent particle approximation (dotted lines) and the BSE (continuous lines) calculated for PMA_2_PbBr_4_ (dark red) and Cs_2_^PMA^PbBr_4_ (dark blue). Both optical absorption spectra are blue-shifted by 0.6 eV in order to match the onset of the experimental optical absorption spectra (gray disks) reported in ref (30). **d.** Energies of bound neutral excitations in PMA_2_PbBr_4_ (left panel) and Cs_2_^PMA^PbBr_4_ (right panel). Bright states are represented with dark red (blue), and dark states are represented in gray. The zero energy level is set to the quasiparticle band gap. Labels “L” and “IL” refer to layer and interlayer states, respectively. **e.** Electron localization probability corresponding to the lowest excited state (L-state) and the ninth excited state (IL-state) when the hole is fixed on a Pb atom in the middle perovskite layer for PMA_2_PbBr_4_.

We calculate electronic and optical excitations of PMA_2_PbBr_4_ within the *GW*+BSE framework, as implemented in the BerkeleyGW code^68−70^ (see the SI for computational details and convergence studies). Using a “single-shot” *G*_0_*W*_0_ approach, employing the Godby-Needs plasmon-pole model and the static remainder approximation^300,400^, and starting from DFT/PBE including SOC, we find a quasiparticle band gap of 3 eV. In Figure 1b, we show the *GW*+BSE optical absorption spectrum, in comparison with the independent particle spectrum and with experiment. The optical spectrum exhibits a sharp peak at the onset of absorption, 467 meV below the quasiparticle band gap, reflecting strong electron–hole interactions. The line shape of our calculated optical absorption spectrum, which includes an empirical 0.1 eV broadening, is in very good agreement with experimental data reported in ref (30), as shown in Figure 1c. However, the position of the main excitonic peak is red-shifted with respect to experiment by 0.6 eV; this discrepancy is in line with previous *G*_0_*W*_0_ calculations reported for 3D perovskites and has been associated with the *G*_0_*W*_0_ starting-point dependence, which can be partially resolved through self-consistency or using hybrid functional starting points.^71−75^ In addition, our convergence studies predict a systematic underestimation of the band gap in the range of 0.1–0.2 eV (see the SI). To reduce computational effort, while capturing the physics, we restrict our level of theory for the quasiparticle energies to the single-shot *G*_0_*W*_0_@PBE approach and use empirical scissor corrections only to compare experimental and computed optical absorption spectra.

In Figure 1d, we show the fine structure of the computed bound excited states, i.e., those below the quasiparticle band gap; of main interest are the first 16 excited states which correspond to transitions between the 4 degenerate conduction and valence band edge states. The first 8 states are grouped in the lower energy region between −0.5 eV and −0.4 eV and correspond to excitons localized within the PbBr_4_ perovskite layer (shown in Figure 1e); we label this group of states with L. These states include three transitions which are optically inactive (dark), and five which are optically active (bright) and contribute to the main excitonic peak of the optical absorption spectra (Figure 1c). The lowest excited state is dark, with an energy approximately 30 meV below the second highest bright state, in very good agreement with the measured fine structure of closely related PEA_2_PbBr_4_ (PEA = phenylethylammonium).^76^ Four of the five bright states are nearly degenerate and optically active for a light polarization direction along the PbBr_4_ plane, while the fifth one (23 meV above) is optically active for light polarized in the direction perpendicular to the PbBr_4_ layer; this is consistent with recent experiments reporting the occurrence of bright in- and out-of-plane excitons in RP perovskites.^64,76−78^ The 8 states in the second group, occurring just above −0.2 eV (Figure 1d), are all nearly degenerate and dark; in these states, photoexcited electrons are localized in a different layer from the holes (An example of one of the eight states is shown in Figure 1e.), and we label these with IL.

Next, we probe the contribution of the PMA cation layer to computed excitons described above by investigating how these properties change in a fictitious scenario when the organic cation, PMA, is replaced by a monatomic cation, Cs, without changing the structure of the PbBr_4_ octahedral layers; this structure is labeled as Cs_2_^PMA^PbBr_4_. As expected, the DFT-PBE band structure of Cs_2_^PMA^PbBr_4_ displays nearly identical band edges to PMA_2_PbBr_4_, since the A-site cations do not contribute directly to these states. Furthermore, optical spectra for Cs_2_^PMA^PbBr_4_ and PMA_2_PbBr_4_ have onsets at very close energies. The quasiparticle band gap and exciton binding energy of Cs_2_^PMA^PbBr_4_ are blue-shifted with respect to PMA_2_PbBr_4_ by 0.2 eV, while the order of the first 16 excited states states discussed above remains qualitatively similar. The difference in the exciton binding energies computed in the two cases correlates with the dielectric constants of the two model layered perovskites (2.8 for PMA vs 2.4 for Cs^PMA^), and the ratio of the binding energies (0.69) is very close to the squared inverse ratio of the high frequency dielectric constants, 0.73, as expected from the hydrogenic model.^79^ This contribution from the PMA cation is in stark contrast to 3D halide perovskites.^35^ It is important to note that our focus in this analysis is on the high frequency (electronic) dielectric screening, and all our calculations ignore the contribution to screening arising from phonons, which has been recently shown to reduce exciton binding energies of semiconductors and insulators by up to 50%,^39^ when the exciton binding energies and the LO phonon energies have similar energies. In *n* = 1 Ruddlesden–Popper perovskites, exciton binding energies can reach values up to 10–20 times larger than characteristic phonon frequencies,^80^ suggesting that the phonon contribution to screening is negligible in this case.

Figure 1 and the arguments above strongly suggest that organic cation layers are important contributors to the dielectric screening of excitations in layered perovskites. To understand this contribution, we perform a systematic analysis of layered perovskites with A-site cations of different sizes and shapes: ethylammonium (EA), butylammonium (BA), phenylmethylammonium (PMA), and naphthalenemethylammonium (NMA). The size of the organic cation determines the interlayer distance, *d*, between the PbBr_4_ layers and the degree of octahedral tilting within each layer, two structural parameters that are expected to impact optoelectronic properties.^30,81^ In the following, we will study the influence of both of these parameters separately, using model and experimental layered perovskite structures.

**Figure 2:**
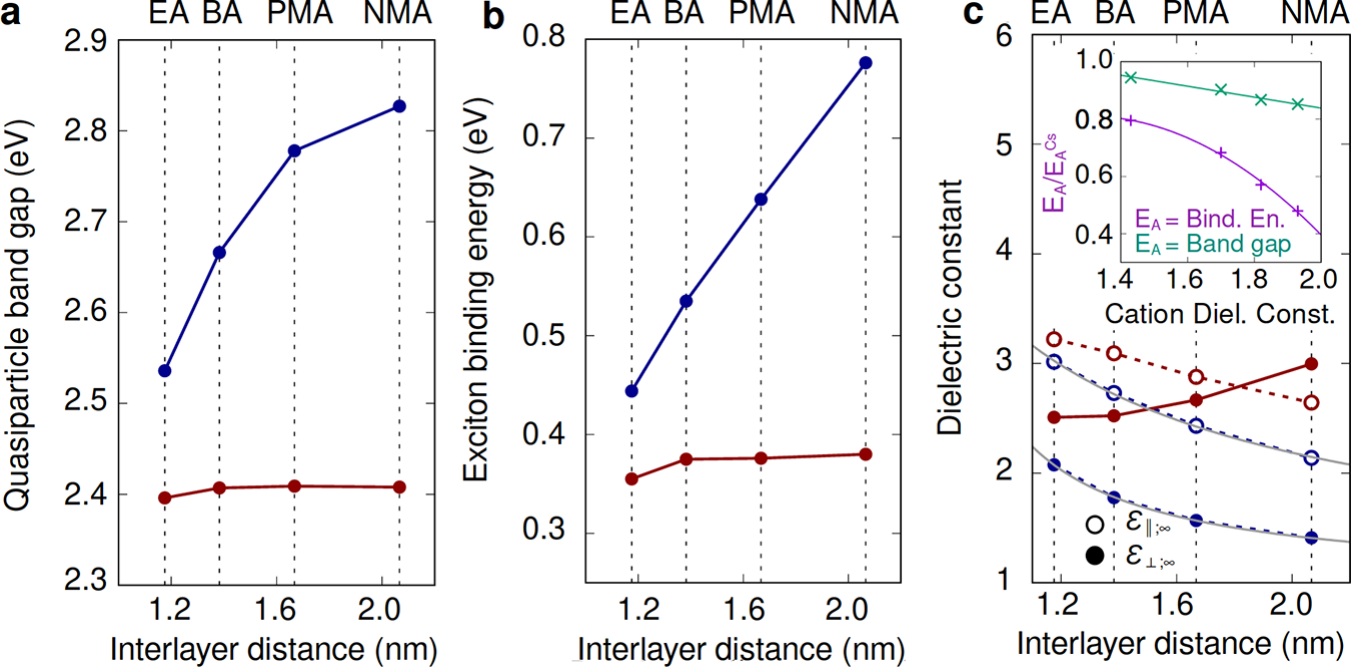
Quasiparticle band gaps (a), exciton binding energies (b), and optical dielectric constants for the in- and out-of-plane polarization directions (c) calculated within the *GW*+BSE framework for model structures without octahedral tilting including organic cations EA, BA, PMA, and NMA (dark red data points) and with Cs replacing the organic cations for the same interlayer distance (dark blue data points). The dark blue and dark red lines in all plots are guides to the eye. The gray lines in (c) correspond to fits of the dielectric constants as a function of the interlayer distance, as described in the main text and SI. The inset of (c) depicts the dependence of the fractional difference between the quasiparticle band gap and exciton binding energy on the cation dielectric constant calculated as described in the main text. The continuous lines in the inset correspond to quadratic and linear fits of the data, as discussed in the main text.

**Table 1:** Cation Layer Dielectric Constants Calculated Using the Dielectric Model Described in the Main Text and the SI (Columns 2–4), and the Clausius-Mosotti Relation (Column 5)

component	ε_⊥_^∞^	ε_∥_^∞^	ε_ave_^∞^	ε_ave;CM_^∞^
EA	1.27	1.52	1.43	1.74
BA	1.53	1.79	1.70	1.94
PMA	1.86	1.81	1.82	2.11
NMA	2.36	1.76	1.93	2.40
inorganic layer	5.81	4.22	4.64	N/A

We first analyze the role of interlayer separation exclusively, by constructing a set of model structures with undistorted PbBr_4_ layers (Pb-Br bond length of 2.937 Å, as in Ref. (500)) which do not exhibit any octahedral tilting, and are separated by inter-layer distances reported in Refs. (30 and 81) and Ref. 3 (see the SI for details); we compare calculations performed on these model structures constructed with the organic cation and with Cs. For this analysis, we perform *GW*+BSE calculations for 8 distinct structures (see the SI for details). As in Figure 1, DFT/PBE band gaps are independent of the interlayer distance or the A-site cation type (see Table S1 of the SI). Similarly, *G*_0_*W*_0_ quasiparticle band gaps and *GW*+BSE exciton binding energies vary by less than 50 meV across the five model structures with different organic A-site cations. On the other hand, upon replacement of the organic A-site cation by Cs, our calculated quasiparticle band gaps increase with an increasing interlayer distance over a range of up to 0.3 eV, as shown in Figure 2a, and exciton binding energies follow the same trend spanning a range of up to 0.4 eV (Figure 2b).

The large difference between band gaps and exciton binding energies for structures with and without the organic cations can be explained from a comparison of the dielectric constants of the two types of structures (Figure 2c). In Figure 2c, we plot both the in-plane and out-of-plane polarization directions, ε_∥_^∞^ and ε_⊥_^∞^, respectively, in order to account for the anisotropy of these systems. In all cases, the A-site cation layer contributes to an increase in the dielectric constants (both in- and out-of-plane) by up to a factor of 2, with the contribution increasing as the interlayer distance increases, thereby reducing the quasiparticle band gap and the exciton binding energies.

The dielectric constants shown in Figure 2c include a contribution from the inorganic layer ε_∥(⊥)_^∞;L^ and the organic cation layer ε_∥(⊥)_^∞;A^. To decouple these contributions from the bulk dielectric constant we calculated within the random phase approximation (RPA),^84,85^ we develop a simple model for the effective dielectric constant, inspired from the elementary formulas for in-series and in-parallel capacitors, equivalent to previous approaches described in the literature^41,64^ (see the SI for details). We first extract the dielectric constantand thickness (*d*_L_) of the PbBr_4_ layer , and the dielectric constant of the cation layer by fitting the model expressions to the dielectric constants obtained for the Cs-based model structures to obtain *d*_L_ = 7.41 Å (close to the width of a PbBr_4_ octahedron of 5.9 Å), ε_⊥_^∞;L^ = 5.81 and ε_∥_^∞;L^ = 4.22 (consistent with 3D lead-bromide perovskites^39^), and ε_⊥(∥)_^∞;A^ ∼ 0.99 (consistent with vacuum permittivity).

Using this simple model, we extract the dielectric constants corresponding to the layers with different cation species given in Table 1. The ratio of exciton binding energies and quasiparticle band gaps calculated for structures with and without organic cations correlates quadratically and linearly with the average dielectric constant of the organic cation layer, respectively, consistent with the assumption that organic cations screen charge carriers in layered halide perovskites (see the inset of Figure 2c). Interestingly, the dielectric constants of the organic cation layers calculated with this fitting procedure follow a very similar trend to those calculated using the Clausius–Mosotti relation^86^ and molecular polarizabilities calculated within the time-dependent density functional perturbation theory framework (TD-DFPT),^82,83^ at the RPA level. The Clausius–Mosotti dielectric constants consistently overestimate first-principles results; we tentatively assign this difference to the absence of effects emerging from small intermolecular interactions or to inaccuracies in estimating the organic layer volume (which does not take into account interpenetration between the inorganic and organic layers). Despite this systematic discrepancy, the agreement between the two independently calculated trends suggests that it may be possible to estimate dielectric constants for the organic cation layers without the need for computationally demanding *GW* calculations.

**Figure 3:**
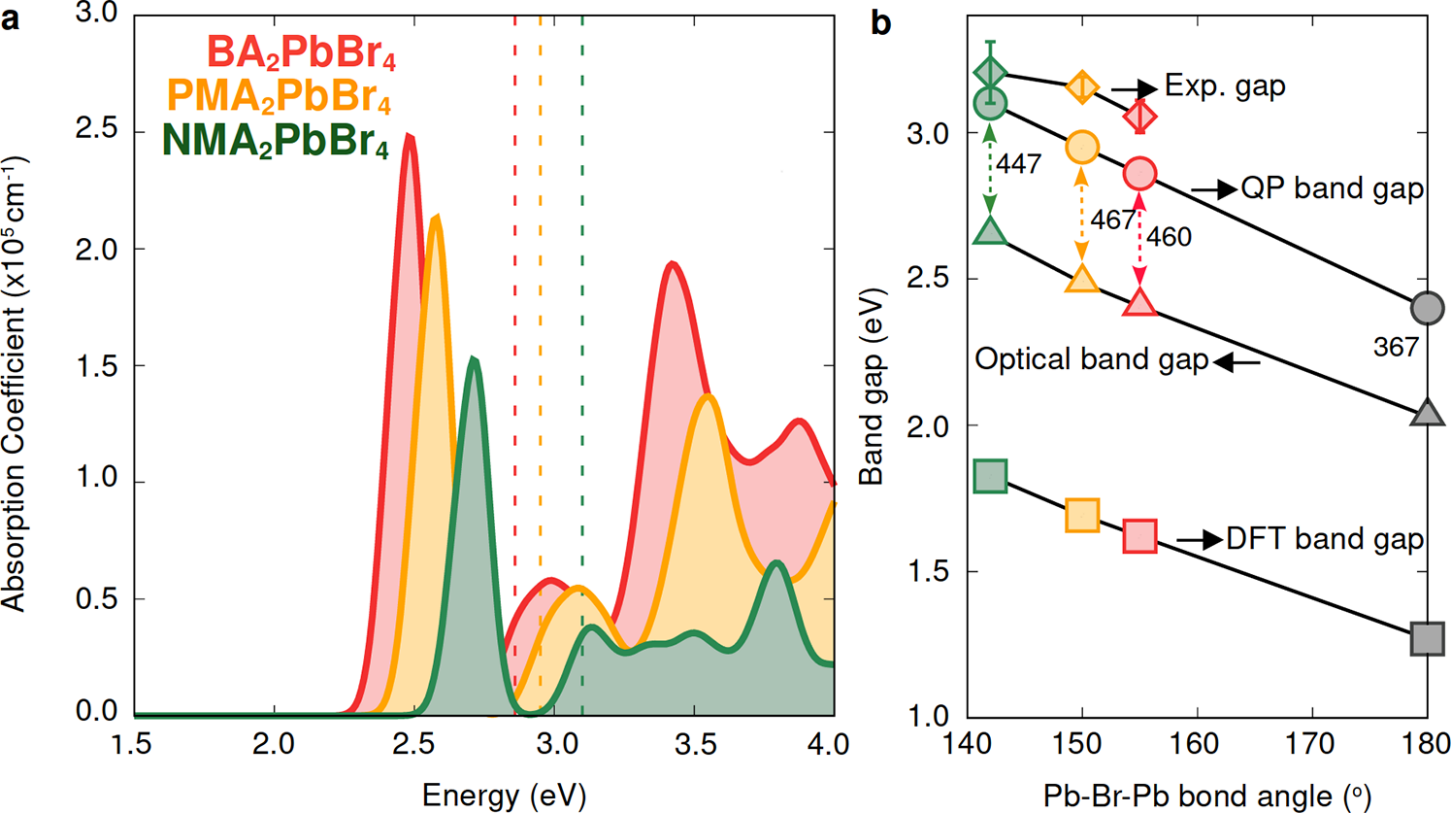
(a) Optical absorption spectra calculated for the experimental structures of BA_2_PbBr_4_ (red), PMA_2_PbBr_4_ (yellow), and NMA_2_PbBr_4_ (green) as described in detail in the SI within the *GW*+BSE approach. Dotted lines mark the quasiparticle band gaps in each case. (b) Comparison between the mean field DFT, optical, quasiparticle, and experimental band gaps for BA_2_PbBr_4_ (red), PMA_2_PbBr_4_ (yellow), and NMA_2_PbBr_4_ (green) and the band gaps calculated for the model untilted layered perovskites (gray data points). The band gaps corresponding to the model layered perovskites are obtained as an average of the band gaps shown in Figure 2. Experimental optical band gaps are read from the positions of the main excitonic peaks in the optical absorption spectra measured at room temperature, as reported in ref (600) for BA_2_PbBr_4_ and ref (30) for PMA_2_PbBr_4_ and averaged over values from refs (30 and 87) for NMA_2_PbBr_4_.

In Figure 3a, we show optical spectra calculated within the *G*W+BSE approach for experimental structures of BA_2_PbBr_4_, PMA_2_PbBr_4_, and NMA_2_PbBr_4_, (see SI for details. (300)) which exhibit increasing Pb–Br–Pb bond angles as the cation size increases (see Figure 3b). Unlike the model case, excitonic peaks blue shift as the size of the cation increases. The same trend is followed by the band gaps (both from DFT-PBE and *G*_0_*W*_0_), with an almost linear correlation with the magnitude of the in-plane Pb–Br–Pb bond angles, in very good agreement with previous computational studies of layered perovskites,^88^ three-dimensional perovskites,^29,89^ and experiment (Figure 2b). In close agreement with Figure 2b, we find that the exciton binding energy is largely independent of the cation type for the three experimental structures analyzed here. Furthermore, exciton binding energies are systematically 20% larger than those obtained for the model structures, in part due to the increase in charge carrier effective masses with increasing octahedral tilting; this effect has been explicitly computed in previous studies both for 3D perovskites^29,89^ as well as in layered perovskites.^36^

Finally, we return to the dielectric model of Figure 2c to illustrate how the choice of the organic cation can tune the dielectric properties of layered perovskites. In Figure 4, we visualize the dependence of the effective dielectric constant in two cases: (i) with *d*_L_ = 7.41 Å and ε_ave_^∞; L^ = 4.64 fixed and (ii) with *d*_A_ = 9.26 Å and ε_ave_^∞; A^ = 1.82 (PMA) fixed. The most effective route to tuning the effective dielectric constant, according to our model, is by modifying the dielectric constant of the organic cation layer; this is in stark contrast to 3D perovskites. For example, doubling the dielectric constant of the PMA layer could increase the effective dielectric constant of a layered perovskite by up to 50% without changing the inorganic layer chemistry. By contrast, scanning through expected dielectric constants of inorganic layers (corresponding to chloride, bromide, and iodide perovskites) we find that the effective dielectric constants are much less sensitive to the dielectric properties and size of the inorganic layer. This indicates that the exciton binding energies of layered perovskites with different halogen compositions should be mostly dictated by the curvature of the conduction and valence band edges and less so by the dielectric properties of the inorganic layer. Our results are in line with recent experimental studies implementing cations which are highly polar,^43^ including heavier ions^44^ or including two ammonium groups;^90^ refs (43 and 44) report a significant reduction in the exciton binding energy, as qualitatively predicted by the simple model depicted in Figure 4.

**Figure 4:**
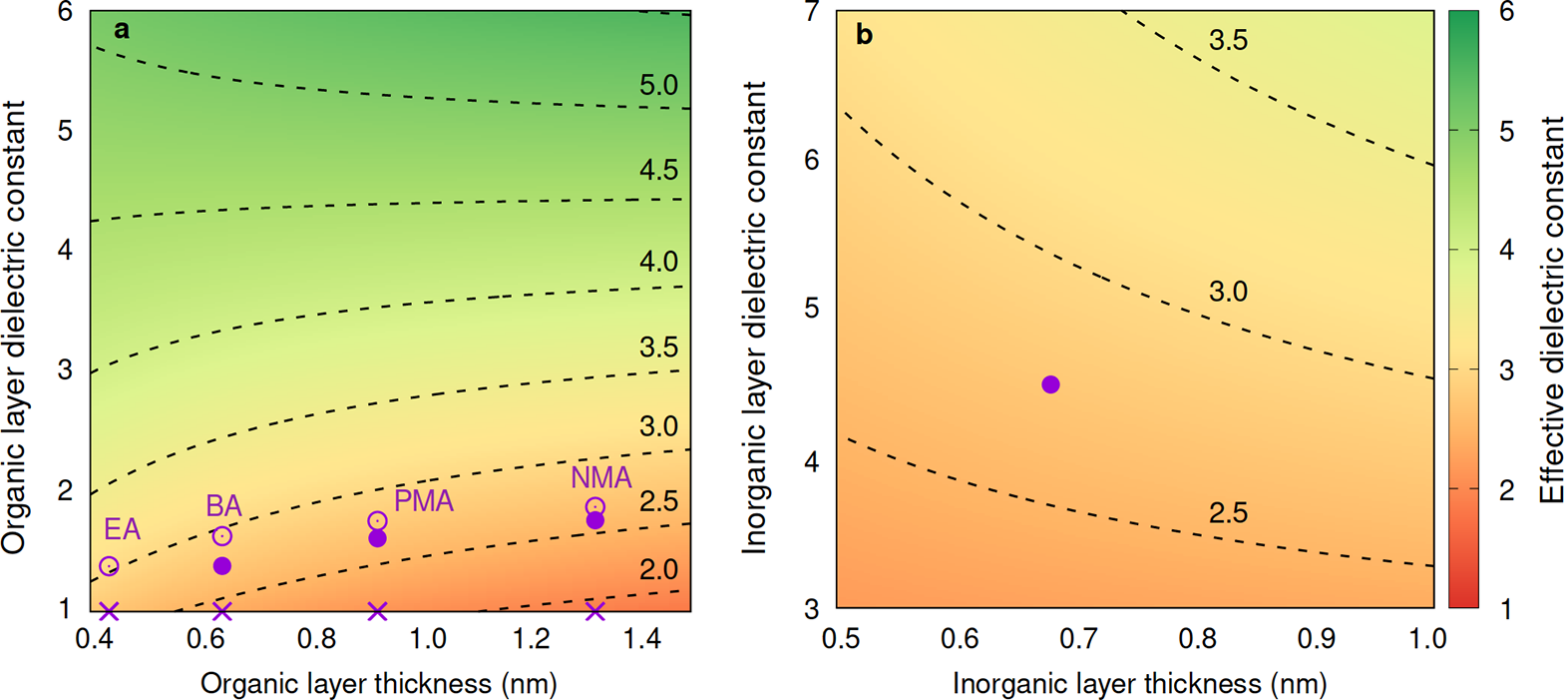
(a) Effective dielectric constant of a hypothetical lead-bromide layered perovskite with the width of the inorganic layer, *d*_L_ = 7.41 Å, and the average dielectric constant of the inorganic layer, ε_ave_^∞;L^ = 4.64, as a function of the width and dielectric constant of the organic layer. The empty circles correspond to calculated values of the model EA_2_PbBr_4_, BA_2_PbBr_4_, PMA_2_PbBr_4_, and NMA_2_PbBr_4_, and the filled disk corresponds to calculated values for the experimental structures of the latter three layered perovskites. The points marked by crosses correspond to dielectric constants calculated for model structures in which the organic cation is replaced by Cs. (b) Effective dielectric constant of a hypothetical layered perovskite with PMA as the organic layer, as a function of the inorganic layer thickness and dielectric constant. The purple disk corresponds to the case of PMA_2_PbBr_4_ (ε_ave_^∞;A^ = 1.82 and an inorganic layer thickness of 7.41 Å). The effective dielectric constant in both (a) and (b) is defined as ε_eff_^–1^ = (2ε_∥_^–1^ + ε_⊥_^–1^)/3, and we assume that dielectric constants of the organic and inorganic layers are isotropic (ε_∥_^∞;A(L)^ ∼ ε_⊥_^∞;A(L)^ ∼ ε_ave_^∞;A(L)^).

In conclusion, we have presented a comprehensive first-principles analysis of the optoelectronic properties of several organic–inorganic Ruddlesden–Popper layered lead-halide perovskites. We have shown that unlike their three-dimensional counterparts, in layered perovskites the organic cations play a key role in the optical properties of these materials, through contributions to the dielectric screening of photogenerated carriers within the layers. Using a set of simplified model structures, we have quantified this contribution, calculated the dielectric constant corresponding to the organic cation layer, and shown that dielectric screening originating with the organic cation counterbalances quantum confinement effects and acts to reduce the exciton binding energy as compared to inorganic monocations. This intuition was confirmed by calculations for experimental structures of BA_2_PbBr_4_, PMA_2_PbBr_4_, and NMA_2_PbBr_4_, which showed that octahedral tilting mainly contributes to shift the position of the optical absorption onset but does not impact the dielectric properties of layered perovskites significantly. Finally, we used a simple model to predict that the effective dielectric constant of layered perovskites can be significantly enhanced by increasing the dielectric constant of the organic cation or decreasing the distance between the inorganic layers. Thereby, the interlayer organic cations could potentially facilitate the reduction of exciton binding energies more efficiently than the chemical composition of the inorganic layers, as previously expected from their 3D counterparts. The chemical intuition outlined in this work can provide a reliable pathway toward the development of efficient optoelectronic devices implementing layered hybrid organic–inorganic halide perovskites.

## Supporting Information Available

Computational and model details, as well as convergence tests and additional references (PDF)
