# Supplementary material for: Screening of Excitons by Organic Cations in Quasi-Two-Dimensional Organic–Inorganic Lead-Halide Perovskites
Source: Nano Lett. 2022 Jun 9;22(12):4870–8. doi: 10.1021/acs.nanolett.2c01306 (PMC9228398; doi:10.1021/acs.nanolett.2c01306)
Supplement: Supplementary file 1 — nl2c01306_si_001.pdf [file nl2c01306_si_001.pdf]

Screening of Excitons by Organic Cations in Quasi-Two Dimensional Organic-Inorganic Lead-Halide Perovskites Supplementary Information

Marina R. Filip,[*,†] Diana Y. Qiu,[‡] Mauro Del Ben,[¶] and Jeffrey B. Neaton[§,‖,⊥]

†*Department of Physics, University of Oxford, Clarendon Laboratory, Oxford, OX1 3PU, United Kingdom*

‡*School of Engineering and Applied Science, Yale University, New Haven, CT 06511, USA*

¶*Computational Science Division, Lawrence Berkeley National Laboratory, Berkeley, CA 94720, USA*

§*Department of Physics, University of California, Berkeley, CA 94720, USA*

‖*Materials Science Division, Lawrence Berkeley National Laboratory, Berkeley, CA 94720, USA*

⊥*Kavli Energy Nano Sciences Institute at Berkeley, Berkeley, CA 94720, USA*

E-mail: marina.filip@physics.ox.ac.uk

Computational Setup

Construction of Model Structures

Here, we refer to the smallest perovskite unit cell with undistorted octahedra as the model structure. Model structures analyzed in Figure 2 of the main manuscript are constructed

and fixed as sheets of perfect $PbBr_6$ octahedra, with Pb-Br bond lengths of 2.937 Å(the same as the Pb-Br bond length found in the cubic phase of the three dimensional perovskite $CsPbBr_3$ measured in Ref. 1). The inter-layer distance is fixed to the measured inter-layer distance in each case, as follows: 11.76 Å for EA_2PbBr_4,[2] 13.83 Å for BA_2PbBr_4,[3] 16.68 Å for PMA_2PbBr_4 and 20.67 Å for NMA_2PbBr_4.[4] The organic cations are placed inside the inter-layer space in such a way that NH_3 groups are closest to the $PbBr_4$ layers, and neighboring molecules along the direction perpendicular to the layer are rotated with respect to each other by 90 degrees; this choice is made so as to best mimic the experimental structures. We obtain the final model structures used in calculations by letting only the molecular cations relax, while keeping the $PbBr_4$ layers and lattice parameters fixed. We proceed in a similar way with constructing the model structures in which Cs replaces the molecular cations.

Ground State Properties: Density Functional Theory Calculations

All mean field calculations are performed within the generalized gradient approximation to DFT, using the Perdew-Burke-Erzerhof parametrization (DFT-PBE),[5] as implemented in the Quantum Espresso code.[6,7] For all calculations we use the (standard) optimized norm-conserving Vanderbilt pseudopotentials[8] of the Pseudo-Dojo database,[9] a plane wave cutoff of 50 Ry, and a shifted $1\times6\times6$ and $1\times4\times4$ for structures without and with octahedral tilting, respectively. We do not include relativistic effects in structural optimizations, however all subsequent electronic structure calculations fully take into account spin-orbit coupling.

DFT/PBE electronic band structures calculated for the model un-tilted structures with Cs generally exhibit a four-fold-degenerate conduction band bottom (CBB) and valence band top (VBT). When the organic cation is included in the model structure, a small splitting of the degeneracy of up to 10 meV appears for EA, BA and PMA. In the case of NMA, this degeneracy breaking is much larger, reaching up to 150 meV. The same differences are seen both for the DFT and *GW* band edges, and we associate them with artefacts of our structural models; indeed, in electronic band structures calculated for the measured experi-

mental structures of BA_2PbBr_4, PMA_2PbBr_4 and NMA_2PbBr_4, this degeneracy splitting is not observed. In order to correct for these artefacts, we average the calculated valence band top and conduction band bottom for all four model structures, which include the organic cation, replacing the calculated values with an average of the energies of the 4 highest occupied and 4 lowest unoccupied electronic states both for the DFT and *GW* band quasiparticle eigenvalues. DFT and QP band gaps reported in Table S1 are calculated in this way.

Table S1: Band gaps calculated at each level of theory for all layered perovskite considered in this work (with model or experimental structure): DFT band gaps, quasiparticle band gaps calculated from G_0W_0, optical band gaps (the lowest excited state energy calculated from BSE). The fifth and ninth columns correspond to exciton binding energies calculated in each case as the difference between the quasiparticle band gap and the energy of the first excited state. All values reported are in eV. The cations labeled as Cs^A correspond to the cases where the A organic cation is replaced by a Cs atom while keeping the same inorganic layer structure as in the A_2PbBr_4

	Model Structure				Experimental Structure			
Cation	DFT	QP	Opt.	Bind. En.	DFT	QP	Opt.	Bind. En.
EA	1.286	2.396	2.041	0.353	N/A	N/A	N/A	N/A
BA	1.273	2.407	2.032	0.367	1.621	2.860	2.400	0.460
PMA	1.283	2.409	2.033	0.367	1.692	2.951	2.484	0.467
NMA	1.284	2.408	2.028	0.380	1.826	3.097	2.650	0.447
Cs^{EA}	1.281	2.536	2.092	0.444	N/A	N/A	N/A	N/A
Cs^{BA}	1.277	2.666	2.131	0.536	N/A	N/A	N/A	N/A
Cs^{PMA}	1.278	2.778	2.140	0.643	1.706	3.170	2.489	0.681
Cs^{NMA}	1.278	2.827	2.051	0.776	N/A	N/A	N/A	N/A

Excited State Properties: GW+BSE calculations

All excited state calculations are performed within the *GW* approximation and the Bethe-Salpeter equation, as implemented in the BerkeleyGW code.[10–12] In particular, quasiparticle energies are calculated using the GPU-CUDA implementation of the BerkeleyGW code, as described in Ref.[12]

Quasiparticle energies are computed within the single-shot G_0W_0 approximation, using the Godby-Needs plasmon pole model to approximate the frequency-dependent dielectric function.[13] In all calculations we employ the static-remainder approximation[14] in order to

facilitate faster convergence with respect to empty electronic states. We converge the quasiparticle band gap and the dielectric constant using a model Cs_2PbBr_4 structure with an interlayer distance of 9.3 Å, without octahedral tilting, and convergence parameters obtained therefrom are extrapolated to all other structures considered in this work. In Figure S1 we show the convergence of the quasiparticle band gap with respect to the polarizability cutoff and number of empty states (a) and the density of the **k**-point grid. In addition to ensuring numerical accuracy, the goal of our convergence studies is to determine the minimal parameter set that yields quasiparticle band gaps within an acceptable numerical accuracy, bearing in mind that experimental structures include up to 5 times more atoms in the unit cell. Given the convergence study shown in Figure S1, we conclude that 200 empty states, a 6 Ry polarizability cutoff and a grid of $6 \times 1 \times 6$ is sufficient to converge the quasiparticle band gap within 0.1-0.2 eV of the converged value, which is a reasonable compromise to make given the size of the unit cells planned for the rest of this study. We note that in all calculations, we use a half-shifted grid for the calculation of the dielectric function, and a Γ-centered grid for the calculation of quasiparticle energies. To ensure the reliability of this choice of setup throughout our study, we also test the convergence of the quasiparticle band gap for a Cs_2PbBr_4 with more than double the interlayer distance, and draw a similar conclusion. The convergence parameters thus determined for Cs_2PbBr_4 are used for all model layered perovskites to extract the trends reported in Figure 2 of the main manuscript. For the experimental structures (which are doubled in size and number of atoms compared to the corresponding model structures) we use the same polarizability cutoff as for the model structure, but use a **k**-point grid of $1 \times 4 \times 4$ and 800 empty states to ensure accuracy. Optical absorption spectra and exciton binding energies are calculated using the Bethe-Salpeter equation (BSE) as implemented in the BerkeleyGW code[10] and described in detail in Refs.[15,16] We follow a similar approach to convergence as described for the quasiparticle band gap. We first converge the exciton binding energy of the model Cs_2PbBr_4 with respect to the density of the fine **k**-point grid, and the number of coarse and fine bands, as shown

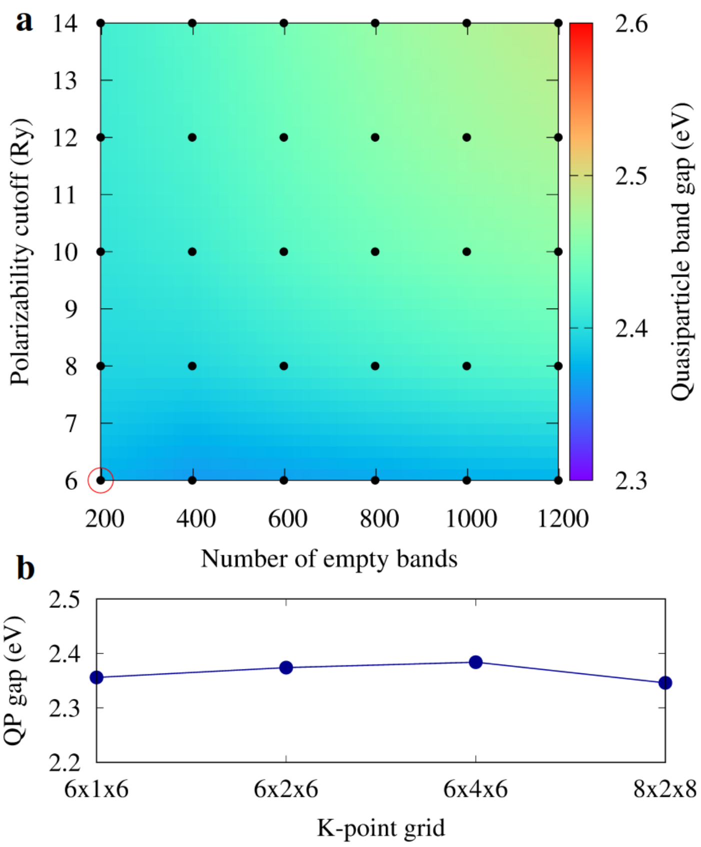

Figure S1: Quasiparticle band gap convergence for a model layered perovskite, Cs_2PbBr_4 with respect to the number of empty states and the polarizability cutoff (a) and with respect to the density of the **k**-point mesh. The map in (a) has been obtained by interpolating through discrete quasiparticle band gap values depicted as black dots on the map. The dot circled in red corresponds to the computational setup used in subsequent quasiparticle band structure calculations for model structures, which we consider as sufficiently converged within the acceptable error-bars set in this study.

in Figure S2. For all model structures we calculate the electron-hole interaction kernel on a $6 \times 1 \times 6$ coarse grid centered at Γ and 10 valence and conduction bands. We then interpolate this kernel using the procedure described in Ref.[15] on a fine grid centered at Γ of $20 \times 1 \times 20$, and 4 valence bands and 8 conduction bands. Based on our convergence studies, we expect this setup to yield exciton binding energies for model structures which are converged within an error-bar of 50 meV. Given the expectation that the band edge shape should change slightly with octahedral tilting, we perform a separate convergence of the exciton binding energy for experimental structures; Figure S3 shows the convergence study for BA_2PbBr_4, as

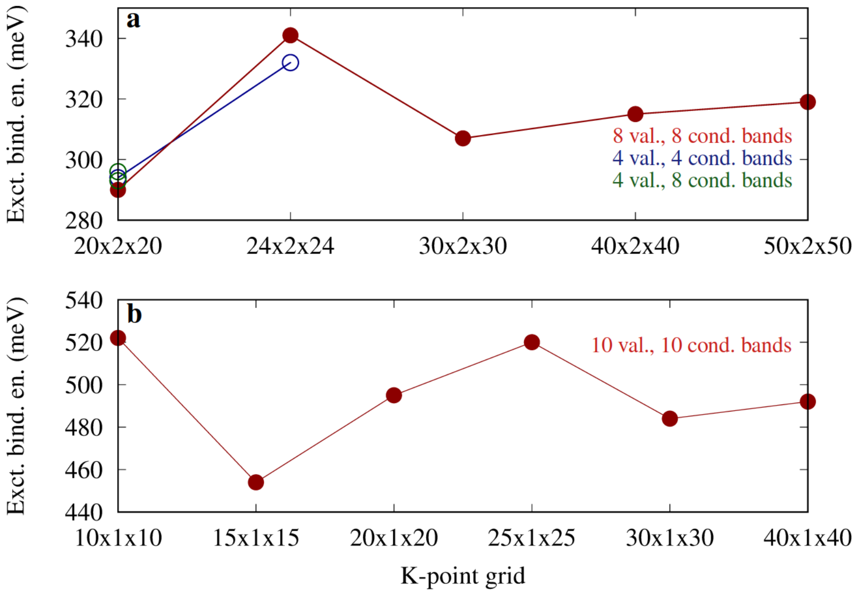

Figure S2: Convergence of the exciton binding energy with respect to the density of the fine **k**-point mesh for a model Cs_2PbBr_4 perovskite (a) and for the experimental structure of BA_2PbBr_4.

a representative case. We find that a Γ centered fine grid of $1 \times 30 \times 30$ should be sufficient to converge the exciton binding energy within an error bar of 50 meV, interpolating the kernel calculated for 20 coarse valence and conduction bands over 10 valence and conduction bands. We use the same setup to also calculate the optical absorption spectra shown in Figure 3 of the main manuscript, for which we use momentum matrix elements rather than velocity matrix elements in order to reduce the computational cost (see Ref. 10 for more details).

As discussed in the main manuscript, the structural anisotropy of layered perovskites is reflected in an anisotropic dielectric function (and constants). In addition, we observe that quasiparticle band gaps and exciton binding energies change by up to 0.1 eV depending on whether we chose the polarization direction for the dielectric constant calculation as in-plane or out-of-plane. This observation is an artefact of the Brillouin zone sampling, and we expect the sensitivity to the polarization direction to reduce as we increase the density of the Brillouin zone sampling. However, in order to mitigate this effect and contain the computational effort, for all calculations performed on the experimental structures we chose

a polarization direction along the crystal $(1, 1, 1)$ direction, while all calculations of exciton binding energy for model structures are performed for an in-plane polarization direction.

Dielectric Constant Model

We extract the dielectric constants corresponding to the organic and inorganic components of the layered perovskite, by modeling the layered perovskite as a dielectric medium in a parallel plate capacitor. When the plates are parallel to the inorganic layers, the electric field inside the capacitor will be perpendicular to the inorganic layer, and the corresponding dielectric constant will be the out-of-plane component $\varepsilon_{\perp}^{\infty}$. Similarly, if the plates are perpendicular, the corresponding dielectric constant will be the in-plane component, $\varepsilon_{\parallel}^{\infty}$. The total capacitance in each case, $C_{\perp(\parallel)}$, is a resultant of individual capacitances of the inorganic and organic layers, which alternate in one direction ad infinitum, connected in series ($C_{\perp}^{\mathrm{L(A)}}$ for $\varepsilon_{\perp}^{\infty;\mathrm{L(A)}}$, Figure S3a) or in-parallel ($C_{\parallel}^{\mathrm{L(A)}}$ for $\varepsilon_{\parallel}^{\infty;\mathrm{L(A)}}$, Figure S3b), leading to:

$$\begin{aligned} \frac{1}{C_{\perp}} &= N\Big(\frac{1}{C_{\perp}^{\mathrm{L}}} + \frac{1}{C_{\perp}^{\mathrm{A}}}\Big), \\ C_{\parallel} &= N\Big(C_{\parallel}^{\mathrm{L}} + C_{\parallel}^{\mathrm{A}}\Big), \end{aligned} \tag{1}$$

where N is the total number of unit cell in the system. For the direction perpendicular to the layers, $C_{\perp} = \varepsilon_{\perp}^{\infty} S_{\perp}/D_{\perp}$, $C_{\perp}^{\mathrm{L}} = \varepsilon_{\perp}^{\infty;\mathrm{L}} S_{\perp}/d_{\mathrm{L}}$ and $C_{\perp}^{\mathrm{A}} = \varepsilon_{\perp}^{\infty;\mathrm{A}} S_{\perp}/d_{\mathrm{A}}$, where $D_{\perp} = N(d_{\mathrm{L}}+d_{\mathrm{A}})$ and $S_{\perp}$ is the total surface of the capacitor plate in Figure S3a. Similarly, for the direction parallel to the layers, $C_{\parallel} = \varepsilon_{\parallel}^{\infty} S_{\parallel}/D_{\parallel}$, $C_{\parallel}^{\mathrm{L}} = \varepsilon_{\parallel}^{\infty;\mathrm{L}} S_{\mathrm{L}}/D_{\parallel}$ and $C_{\parallel}^{\mathrm{A}} = \varepsilon_{\parallel}^{\infty;\mathrm{A}} S_{\mathrm{A}}/D_{\parallel}$, with $S_{\parallel} = N(S_{\mathrm{L}}+S_{\mathrm{A}})$ and $D_{\parallel}$ the distance between the capacitor plates in Figure S3b. Using these expressions and the formulae in Eq. 1, we find the expressions for the in- and out-of-plane dielectric constants reported in the main manuscript:

$$\varepsilon_{\perp}^{\infty} = \frac{d}{d_{\mathrm{L}}/\varepsilon_{\perp}^{\infty;\mathrm{L}} + (d - d_{\mathrm{L}})/\varepsilon_{\perp}^{\infty;\mathrm{A}}}, \tag{2}$$

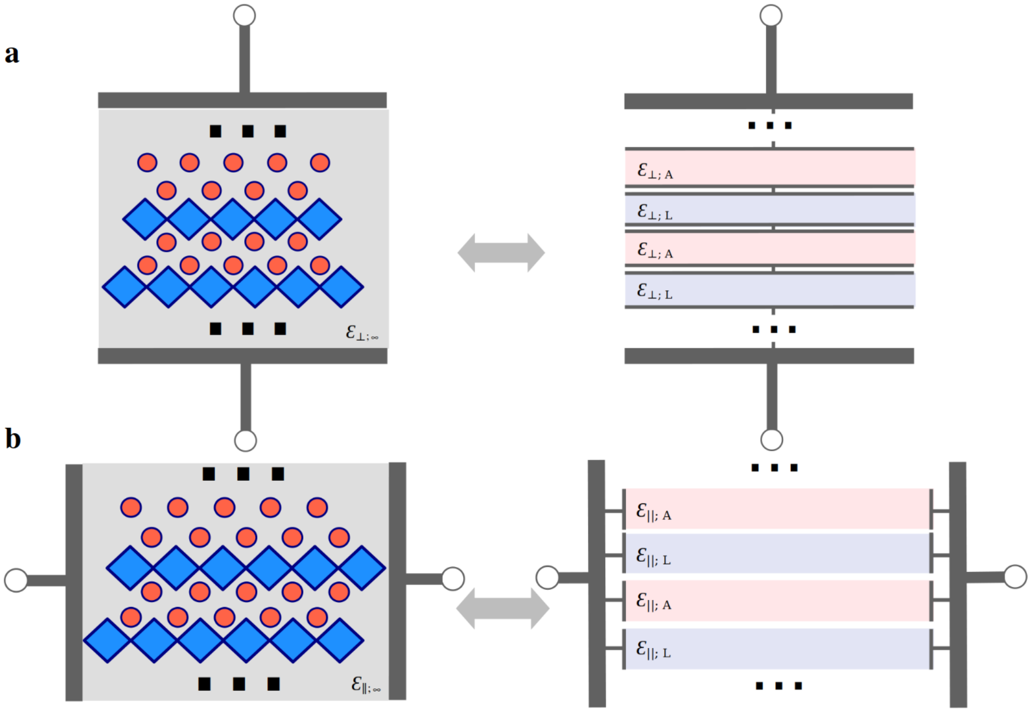

Figure S3: Schematic depiction of the model used to estimate the out-of- (a) and in-plane (b) dielectric constants of the inorganic and organic layers of a layered perovskite.

$$\varepsilon_{\parallel}^{\infty} = \frac{\varepsilon_{\parallel}^{\infty;\mathrm{L}} d_{\mathrm{L}} + \varepsilon_{\parallel}^{\infty;\mathrm{A}}(d - d_{\mathrm{L}})}{d}. \tag{3}$$

To calculate the dielectric constant of the inorganic layer, $\varepsilon_{\parallel(\perp)}^{\infty;\mathrm{L}}$, we first use Eq. 2 to fit the out-of-phase dielectric constants, $\varepsilon_{\perp}^{\infty}$, calculated for the model structures in which Cs replaces the organic cation, as a function of inter-layer distance, d, and obtain $d_{\mathrm{L}} = 7.41$ $\mathring{A}$, $\varepsilon_{\perp}^{\infty;\mathrm{Cs}} \sim 1$ and $\varepsilon_{\perp}^{\infty;\mathrm{L}} = 5.81$. Second, we use Eq. 3 and the parameter d_{L} already calculated to fit the in-plane dielectric constants calculated for the model structures in which Cs replaces the organic cation to obtain $\varepsilon_{\parallel}^{\infty;\mathrm{A}} \sim 1$ and $\varepsilon_{\parallel}^{\infty;\mathrm{L}} = 4.22$. With $\varepsilon_{\parallel(\perp)}^{\infty;\mathrm{L}}$ and d_{L} thus calculated then we use the formulae in Eqs. 2 and 3 to calculate $\varepsilon_{\parallel(\perp)}^{\infty;\mathrm{A}}$ for each organic molecule, as reported in the main manuscript. We note in passing that in principle the fitting procedure could start with Eq. 3 followed by Eq. 2, however we have found empirically that this sequence of steps yielded more numerically stable and physically meaningful fitting parameters without having to impose any *a priori* constraints.

Clausius-Mosotti Relation

In Table 1 of the main manuscript we compare dielectric constants of the organic layer calculated using the model described above with values calculated usins the Clausius-Mosotti relation,[17] $\frac{\varepsilon-1}{\varepsilon+2}=\frac{4\pi}{3}\frac{N}{V}\frac{\alpha}{4\pi\epsilon_0}$, where N is the number of molecules in a volume V (in this case the unit cell volume less the volume occupied by the two inorganic layers in the unit cell, $a^2(c-2a)/2$, where a is the in-plane lattice parameter and c is the out of plane lattice parameter), α is the molecular polarizability and ϵ_0 is the permitivity of vacuum. We calculate the components of the polarizability tensor at 0 frequency for each isolated molecular cation in a large cubic box with a side of 30 Å, using the density functional perturbation theory as implemented in the Quantum Espresso package[7] and described in Refs.[18,19] We then obtain α by averaging the diagonal elements of the polarizability tensor and calculate the dielectric constants reported in Table S1, corresponding to the organic layers using the Clausius-Mosotti relation.

References

(1) Möller, C. K. Crystal Structure and Photoconductivity of Cæsium Plumbohalides. *Nature* **1958**, *182*, 1436.

(2) Luo, B.; Guo, Y.; Li, X.; Xiao, Y.; Huang, X.; Zhang, J. Z. Efficient Trap-Mediated Mn^{2+} Dopant Emission in Two Dimensional Single-Layered Perovskite $(CH_3CH_2NH_3)_2PbBr_4$. *J. Phys. Chem. C* **2019**, *123*, 14239–14245.

(3) Sheikh, T.; Nag, A. Mn Doping in Centimeter-Sized Layered 2D Butylammonium Lead Bromide (BA2PbBr4) Single Crystals and Their Optical Properties. *J. Phys. Chem. C* **2019**, *123*, 9420–9427.

(4) Du, K.; Tu, Q.; Zhang, X.; Han, Q.; Liu, J.; Zauscher, S.; Mitzi, D. B. Two-Dimensional

Lead (II) Halide-Based Hybrid Perovskites Templated by Acene Alkylamines: Crystal Structures, Optical Properties and Piezoelectricity. *Inorg. Chem.* **2017**, *56*, 9291–9302.

(5) Perdew, J. P.; Zunger, A. Self-Interaction Correction to Density-Functional Approximations for Many-Electrons Systems. *Phys. Rev. B* **1981**, *23*, 5048.

(6) Gianozzi, P., et al. QUANTUM ESPRESSO: a modular and open-source software project for quantum simulations of materials. *J. Phys.: Condens. Matter.* **2009**, *21*, 395502.

(7) Giannozzi, P. et al. Advanced capabilities for materials modelling with Quantum ESPRESSO. *Journal of Physics: Condensed Matter* **2017**, *29*, 465901.

(8) Hamann, D. R. Optimized norm-conserving Vanderbilt pseudopotentials. *Phys. Rev. B* **2013**, *88*, 085117.

(9) van Setten, M. J.; Giantomassi, M.; Bousquet, E.; Verstraete, M. J.; Hamann, D. R.; Gonze, X.; Rignanese, G.-M. The PseudoDojo: Training and Grading a 85 Element Optimized Norm-Conserving Pseudopotential Table. *Comput. Phys. Commun.* **2018**, *226*, 39–54.

(10) Deslippe, J.; Samsonidze, G.; Strubbe, D. A.; Jain, M.; Cohen, M. L.; Louie, S. G. BerkeleyGW: A massively parallel computer package for the calculation of the quasiparticle and optical properties of materials an nanostructures. *Comp. Phys. Commun.* **2012**, *183*, 1269–1289.

(11) Del Ben, M.; da Jornada, F. H.; Canning, A.; Wichmann, N.; Raman, K.; Sasanka, R.; Yang, C.; Louie, S. G.; Deslippe, J. Large-Scale GW Calculations on Pre-Exascale HPC Systems. *Comp. Phys. Commun.* **2019**, *235*, 187–195.

(12) Del Ben, M.; Yang, C.; Li, Z.; da Jornada, F. H.; Louie, S. G.; Deslippe, J. Accelerating Large-Scale Excited-State GW calculations on Leadership HPC systems. *Proc. of the*

2020 SC20: International Conference for High Performance Computing, Networking, Storage and Analysis (SC) **2020**, *1*, 1–11.

(13) Godby, R. W.; Needs, R. Metal-Insulator Transition in Kohn-Sham Theory and Quasiparticle theory. *Phys. Rev. Lett.* **1989**, *62*, 1169.

(14) Deslippe, J.; Samsonidze, G.; Jain, M.; Cohen, M. L.; Louie, S. G. Coulomb-hole summations and energies for GW calculations with limited number of empty orbitals: A modified static remainder approach. *Phys. Rev. B* **2013**, *87*, 165124.

(15) Rohlfing, M.; Louie, S. G. Electron-Hole Excitations in Semiconductors and Insulators. *Phys. Rev. Lett.* **1998**, *81*, 2312.

(16) Rohlfing, M.; Louie, S. G. Electron-Hole Excitations and Optical Spectra from First Principles. *Phys. Rev. B* **2000**, *62*, 4927.

(17) Ashcroft, N. W.; Mermin, N. D. *Solid State Physics*; Brooks/Cole, 1976.

(18) Malcioglu, O. H.; Gebauer, R.; Rocca, D.; Baroni, S. turboTDDFT – A code for the simulation of molecular spectra using the Liouville–Lanczos approach to time-dependent density-functional perturbation theory. *Comput. Phys. Commun.* **2011**, *182*, 1744.

(19) Ge, X.; Binnie, S. J.; Rocca, D.; Gebauer, R.; Baroni, S. turboTDDFT 2.0-Hybrid functionals and new algorithms within time-dependent density-functional perturbation theory. *Comput. Phys. Commun.* **2014**, *185*, 2080.
